# Hot electron enhanced photoemission from laser fabricated plasmonic photocathodes

**DOI:** 10.1515/nanoph-2023-0552

**Published:** 2023-10-17

**Authors:** Miguel Martinez-Calderon, Baptiste Groussin, Victoria Bjelland, Eric Chevallay, Valentin N. Fedosseev, Marcel Himmerlich, Pierre Lorenz, Alejandro Manjavacas, Bruce A. Marsh, Holger Neupert, Ralf E. Rossel, Walter Wuensch, Eduardo Granados

**Affiliations:** CERN, European Organization for Nuclear Research, 1211 Geneva, Switzerland; Department of Physics, NTNU–Norwegian University of Science and Technology, NO-7491 Trondheim, Norway; Department of Ultra-Precision Surfaces, Leibniz Institute of Surface Engineering (IOM), Permoserstr. 15, 04318 Leipzig, Germany; Instituto de Óptica (IO-CSIC), Consejo Superior de Investigaciones Científicas, 28006 Madrid, Spain

**Keywords:** hot electrons, plasmonics, photoemission, accelerators

## Abstract

Photocathodes are key elements in high-brightness electron sources and ubiquitous in the operation of large-scale accelerators, although their operation is often limited by their quantum efficiency and lifetime. Here, we propose to overcome these limitations by utilizing direct-laser nanostructuring techniques on copper substrates, improving their efficiency and robustness for next-generation electron photoinjectors. When the surface of a metal is nanoengineered with patterns and particles much smaller than the optical wavelength, it can lead to the excitation of localized surface plasmons that produce hot electrons, ultimately contributing to the overall charge produced. In order to quantify the performance of laser-produced plasmonic photocathodes, we measured their quantum efficiency in a typical electron gun setup. Our experimental results suggest that plasmon-induced hot electrons lead to a significant increase in quantum efficiency, showing an overall charge enhancement factor of at least 4.5 and up to 25. A further increase in their efficiency was observed when combined with semiconductor thin-films deposited over the laser processed surfaces, pointing at potential pathways for further optimization. We demonstrate that simple laser-produced plasmonic photocathodes outperform standard metallic photocathodes, and can be directly produced *in-situ* at the electron gun level in vacuum environments and without any disruptive intervention. This approach could lead to unprecedented efficient and continuous operation of electron sources, and is useful in many applications across scientific disciplines requiring high average and peak current electron beams.

## 1 Introduction

Advanced electron accelerators capable of producing high peak and average currents are used in multitude of applications across technical and scientific fields. Among them, recent progress in FLASH cancer therapies [1, 2] or the development of ultra-high photon flux extreme ultraviolet (EUV) and X-ray sources [3–6] are limited to some extent by the available photocathode technology. Metallic photocathodes are a well-established technology but tend to have relatively low quantum efficiency (QE). This cascades into demanding requirements for ultrafast laser average power at short wavelengths, often in the watts-level and in the deep ultraviolet (DUV) range. Since the first demonstrations of high QE caesium telluride (Cs_2_Te) photocathodes at CERN in the early 90s [7], different semi-conductor materials have been proposed and utilized [8–13]. However, their environmental sensitivity due to a high chemical reactivity results in a limited lifetime, requiring frequent replacements, and imposing ultra-high-vacuum (UHV) conditions for reliable short-term operation.

Finding new routes for enhancing the photo-emissive properties of materials without compromising their reliability is a crucial task. In this regard, plasmonic effects are poised to play a key role in the efficiency of photo-emissive devices [14], including sensors [15], devices based on metamaterials [16, 17], ultrafast photodetectors [18–20], solar energy harvesters [21, 22], or nanoscale light-to-heat transducers [23]. Plasmonic nanostructures such as nanostars [24, 25], nanorods [26], carbon nanotubes [27] and other multi-resonant nanostructures [28–30] have been proposed for electron beam production, with peculiar phase space and spatio-temporal properties. In the field of accelerator physics, plasmonic nanostructures enhancing non-linear and multi-photon photoemission from metallic nanoholes and nanogrooves have been demonstrated [31–33]. This in turn allowed the use of longer wavelength lasers in the near-IR, but at the cost of operating close to the material damage threshold.

There are significant advantages of operating in the linear photoemission regime while exploiting plasmonic effects; the lower intensity allows for higher charge production without material damage, while DUV laser technology currently could allow to operate at high average current. Here, in contrast to non-linear photoemission, the highly localized confinement of surface plasmons can lead to the production of high energy electrons via non-radiative decay, a process characterized by a significant deviation from the Fermi–Dirac distribution traditionally assumed in classic photoemission models. Not surprisingly, this approach has been explored for photovoltaic [34], photochemical [35], and photodetector applications [36, 37]. Moreover, the combination of metallic nanostructures with ultra-thin-film dielectric coatings can further enhance the emission current density and corresponding QE of plasmonic nanostructures [38].

In this work, we propose to enhance the electron yield from copper photocathodes using plasmon-induced hot electrons in laser-fabricated nanostructures. Our experiments come in several steps: First, we show a simple and efficient laser-based fabrication technique for the generation of plasmonic nanostructures in copper, which are resonant under DUV illumination. Second, the copper work function can be lowered by depositing a caesium and tellurium (Cs_*x*_Te_*y*_) thin film over the nanostructures, which amplifies further the charge production. Third, we experimentally demonstrate a charge production enhancement of about 5–25 times from nanostructures compared to a flat copper surface in a DC electron gun driven by nanosecond DUV pulses.

## 2 Theory

Traditionally, metallic nanostructures have been utilized to enhance ultrafast driving optical fields via localized surface plasmon resonances, prompting electron emission in the so-called strong-field regime. In this case, tunnelling and multi-photon effects aid the photoemission process thanks to the resulting infrared optical electric fields exceeding the GV/m barrier [18, 28, 32, 39]. To reach this field level the use of ultrafast or even single-cycle light pulses was mandatory. In contrast, the production of hot electrons via plasmonic resonances in metallic nanoparticles can be, in principle, achieved employing orders-of-magnitude lower laser electric fields and in a quasi-stationary manner [40, 41]. Here, the hot electron distribution arising from the plasmonic interaction can increase the electron emission probability, enhancing the overall quantum efficiency [42, 43]. It should be noted that the resulting electron beam emittance can be degraded due to the resulting broad distribution of photoemitted electron momenta [44], and in particular when using photons at DUV wavelengths. Nevertheless, for applications requiring efficient high charge and average current electron beams, this approach is interesting.

The energy and the corresponding excited-state distribution of hot electrons can be estimated by employing a jellium model in combination with Fermi’s golden rule [37, 45]. This simple approach assumes a non-interacting electron gas confined under a uniform background potential. Upon excitation of the system with light of frequency *ω*, the probability per unit time Γ_*e*_(*E*_*f*_, *ω*) of generating a hot electron in a particular *f* state can be calculated using Fermi’s golden rule [37, 45]. This probability scales directly with the transition matrix elements *M*_*fi*_ and *M*_*if*_, which can be calculated by computing the integral *M*_*fi*_ = *∫V*(**r**, *ω*)*ρ*_*fi*_(**r**)d**r**. Here, *V*(**r**, *ω*) is the induced electric potential arising from the optical excitation of the plasmon, while $\rho_{fi}\left(r\right)=e\Psi_{f}^{*}\left(r\right)\Psi_{i}\left(r\right)$ corresponds to the creation of the two excited carriers (a hole in state Ψ_*i*_ and an electron in state Ψ_*f*_).

We can estimate an upper limit for the resulting quantum efficiency of the photoemission process by computing the number of hot electrons with energy above the work function *W* that are generated per absorbed photon. This quantity is related to the figure of merit $N_{e}\left(\varepsilon \right)$ proposed in [45], which accounts for the hot electrons generated per each plasmon excited in the system that have an energy *E*_*f*_ larger than a defined threshold *ɛ*. In our case this threshold *ɛ* = *W*, resulting in the equation:

(1) $$QE\leq N_{e}\left(W\right)=\frac{\hbar \omega \underset{E_{f}>W}{\sum }\Gamma_{e}\left(E_{f},\omega \right)}{P_{\mathrm{abs}}}.$$

Here, *P*_*abs*_ = *C*_*abs*_ *I*_0_ represents the power absorbed by the nanostructure, which is given by the product of its absorption cross section *C*_*abs*_ and the intensity of the incident illumination *I*_0_.

**Figure 1:**
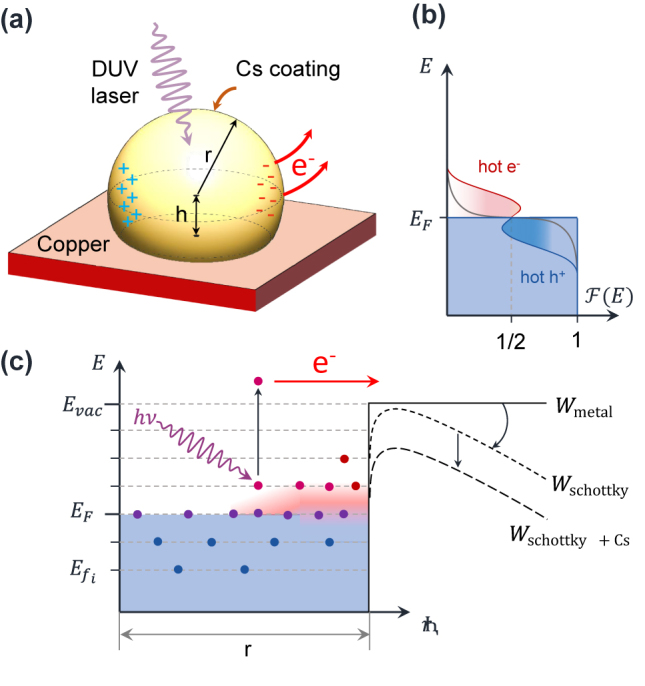
Enhancement of photoemission due to hot carrier generation in plasmonic nanoparticles. (a) Schematic view of a Cu nanoparticle covered with a Cs_*x*_Te_*y*_ thin-film layer illuminated by DUV photons. (b) Schematic of hot-carrier generation: plasmonic nonradiative decay produces electron-hole pairs resulting in a non-equilibrium distribution of hot electrons and holes. Hot electrons are represented by the red areas above the Fermi energy *E*_*F*_. (c) Schematic diagram of the work function reduction induced by the applied field and the addition of a Cs_*x*_Te_*y*_ layer.

Despite the simplicity of this theoretical description, which technically describes the hot electron generation process right after the decay of the plasmon, it provides valuable insight for the design of the photocathode. In particular, it tells us that: (i) The geometry of the selected nanostructure should be such that the induced electric potential *V*(**r**, *ω*) is maximal. (ii) For a fixed QE, the absorption cross section should be as high as possible to maximize the generated current. (iii) The range of the summation in eq. (1) can be maximized by lowering the work function *W*, which can be achieved by employing monolayers of suitable materials such as Cs as depicted in Figure 1(a). We assume that such a thickness of Cs does not affect the optical performance of the nanostructures. Figure 1(b) and (c) summarize the effects involved in the efficiency of the photoemission process.

## 3 Nanostructure laser fabrication

In terms of nanofabrication, it is challenging to simultaneously optimize all these effects for a given nanostructure morphology and spatial distribution, and some techniques trade-off between nanostructure size and inter-spacing with a view on optimizing simultaneously photon absorption and plasmonic resonance at the wavelength of interest. Common approaches include sophisticated nanofabrication techniques such as electron beam lithography (EBL), colloidal deposition, or focused ion beam lithography (FIB) [46]. While these methods feature very high mesoscopic accuracy, they tend to be complex, require multiple fabrication steps and take significant time to cover mm^2^ areas. Also, the possibility of *in-situ* and in vacuum photocathode rejuvenation would suppose a great added value for electron guns in accelerator facilities.

To address this nanofabrication challenge, we take an alternative route based on surface nanostructuring via copper nano-ablation using ultrafast pulsed lasers. When the laser fluence is precisely tuned slightly above the material’s ablation threshold, the process leads to two main types of nanofeatures. First, ripple-like sub-wavelength nanostructures (also known as laser induced periodic surface structures, LIPSS) depending on the irradiation wavelength, fluence, number of pulses or wavefront [47–51]. Second, the production of nanoparticle clusters in the range of few tens to hundreds of nanometers arising from the rapid expansion and cooling of the ablation plasma plume generated after the absorption of high intensity laser pulses [52–57]. Moreover, the nanoparticle size and distribution can be controlled to some extent by tuning the laser and fabrication parameters [58].

**Figure 2:**
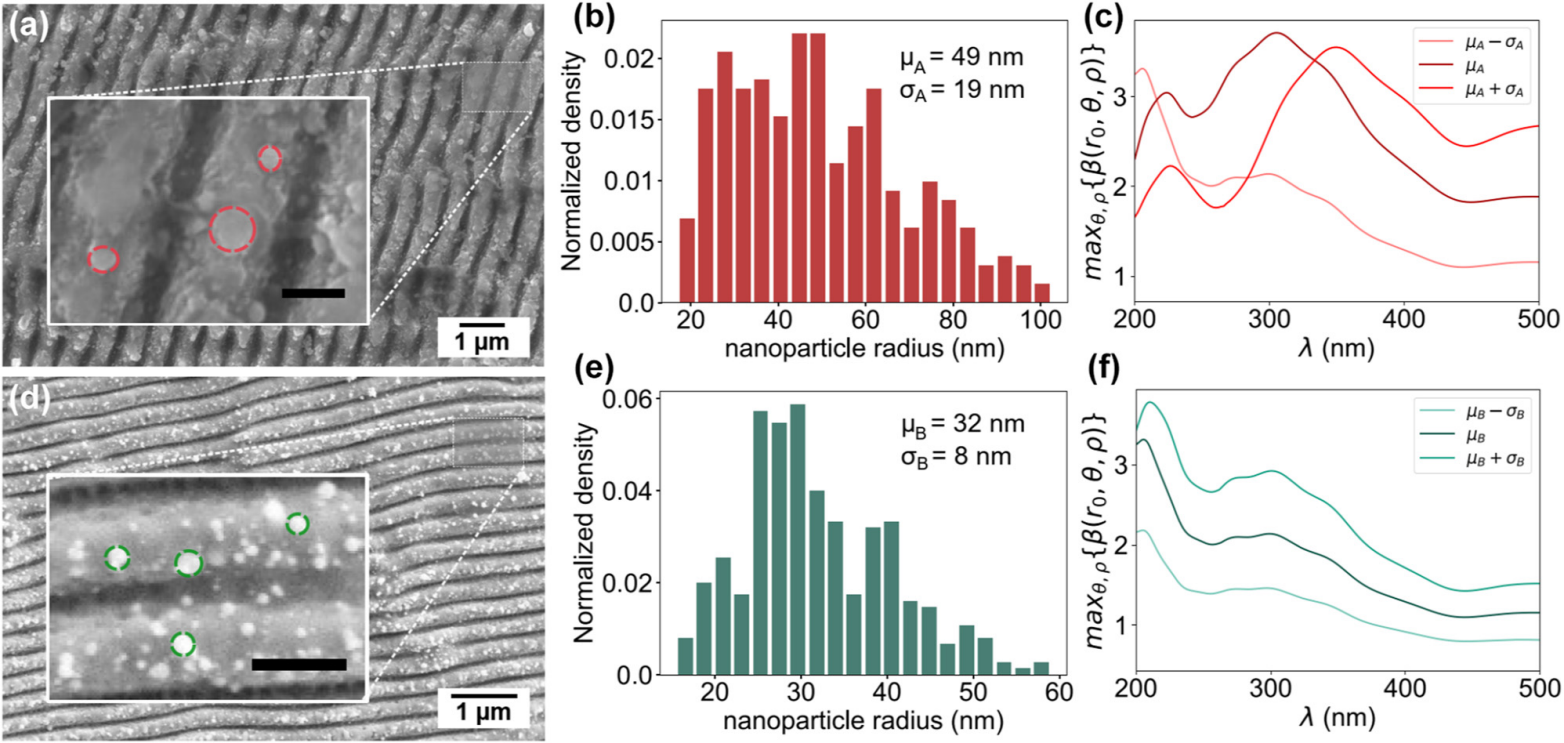
Nanoparticle size and distribution in laser fabricated plasmonic photocathodes. (a, d) SEM images of the nanostructured areas in photocathode (A) and (B) respectively. The images show the structures combining ripple-like patterns with the presence of multiple nanoparticles. Insets show higher magnification images from which the nanoparticles shapes and distribution can be distinguished and some examples of the particles measurements performed. The scale bars correspond to 300 nm in both cases. (b, e) Distribution of the measured nanoparticle radii in photocathode (A) and (B) respectively, showing average radius of 49 ± 19 nm and 32 ± 8 nm. (c, f) Maximum field enhancement max{*β*(*r*_0_, *θ*, *ρ*)} as function of the excitation wavelength for the nanoparticle sizes and distribution measured in photocathode (A) and (B).

We fabricated two photocathodes with different ultrafast laser systems and sets of fabrication parameters. First, photocathode (A) was fabricated using 130 fs pulses at 800 nm wavelength and at 1 kHz repetition rate, leading to the topographies shown in Figure 2(a). Here, the average nanoparticle dimensions (assumed to be quasi-spherical) presented a radius of 49 ± 19 nm (see Figure 2(b)). The nanostructured areas of photocathode (A) consisted in two 2 × 2 mm^2^ squares centered in the photocathode plug, as it is shown later in the article in the inset of Figure 4(a). Second, photocathode (B) was processed using 260 fs pulses at 515 nm with a 100 kHz pulse repetition rate. The resulting surface topography is shown in Figure 2(d), with a statistical nanoparticle size of radius 32 ± 8 nm (see Figure 2(e)). The nanostructured areas consisted of 2 × 2 mm squares distributed along the whole photocathode (B) surface with a checkerboard design, as shown also in the inset of Figure 4(b). It is important to remark that, in the case of photocathode (A), only the central area of the photocathode was treated, having large flat areas that allowed a direct comparison between nanostructures and untreated areas. The checkerboard design in cathode (B) was intended to allow an estimate of the differential QE across the entire surface of the cathode, but the spatial resolution of our experimental setup (about 1.5–2 mm) prevented this measurement to be realized with enough accuracy to resolve the pattern. Both cathodes presented ripple-like nanostructures with a spatial periodicity of 570 nm and 380 nm respectively although these do not significantly alter the local electric field when illuminated with DUV light at 266 nm. Further information as well as simulations of the field enhancement in the produced photocathodes can be found in the Supplementary Material.

With a view on setting upper and lower bounds for the potential field enhancement of these nanoparticles under DUV illumination, we conducted a series of electromagnetic simulations using COMSOL™ in the frequency domain. We simulated copper nanoparticles of variable radius *r* placed on top of a flat copper surface with a penetration depth of 0.2*r* (corresponding to *h* = 0.8*r*) as shown in Figure 1(a). We apply an electromagnetic wave at normal incidence, of wavelength *λ* = 266 nm, and linearly polarized so that the input electric field is parallel to the copper surface plane resembling the experiments. The simulated nanoparticles exhibit clear plasmonic behavior with different resonance peaks of field enhancement depending on the nanoparticle size and excitation wavelength.

We then calculate the field enhancement factor *β* = |*E*|/|*E*_0_| at the nanoparticle surface (*r* = *r*_0_) in spherical coordinates {*r*_0_, *θ*, *ρ*} and computed its maximum max{*β*(*r*_0_, *θ*, *ρ*)} over the entire nanoparticle surface for a range of radius and illumination wavelengths. This data allows to estimate the optimal nanoparticle radius to achieve maximum field enhancement. Figure 2(c, f) show max{*β*(*r*_0_, *θ*, *ρ*)} as a function of impinging wavelength for the nanoparticles of size corresponding to the statistical distribution measured for photocathode (A) and (B), respectively. The simulation results show that when illuminated in the DUV range (around 260 nm), the field enhancement factor *β* of the statistically measured nanoparticles is in the range of 2–3 at the nanoparticle surface.

Although there is a direct connection between the field enhancement and the generation of hot electrons, it is difficult to predict *a-priori* which photocathode could perform better due to the non-linear dependency of Γ_*e*_ with the induced potential at each *r*. For instance, photocathode (A) has an average particle size of *μ*_*r*_ ≈ 49 nm, well matched to the theoretical maximum plasmonic resonance, but with a larger spread *σ*_*A*_ = 19 nm than photocathode (B) *σ*_*B*_ = 8 nm. Additionally, the use of the maximum *β* to assess the QE of the nanoparticle can render inaccurate results since the produced photo-current density should be computed by integrating the electron emission from the entire nanostructure surface and not just at the maximum. Consequently, the predictions shown here are supporting the experimental results only qualitatively.

## 4 Experiments

**Figure 3:**
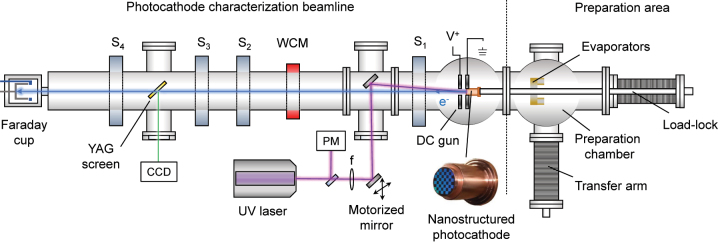
Schematic layout showing the relevant devices of the DC photoelectron gun setup used in the experiments. Elements in the left comprise the photocathode characterization beamline. Items in the right constitute the photocathode preparation area. S_1_ – S_4_ are solenoids, WCM, Wall current monitor; *V* ^+^, positive voltage (anode) in the DC gun; PM, on line power meter.

The photoemission experiments were conducted at the CERN photocathode fabrication facility, which is equipped with a preparation area and a characterization beamline as shown in Figure 3. The nanostructured photocathodes were first inserted into the preparation area fully opened to air atmosphere. This chamber had to undergo a bake-out (heating elements to temperatures from 150° to 250°) to assure proper vacuum conditions for the later transfer to the DC gun characterization beamline (already at UHV and separated by a UHV stainless steel mechanical valve). Further details about the facility and DC gun setup are provided in the Supplementary Material. During the bake-out procedure, Cs and Te were evaporated from the hot surfaces of the deposition chamber resulting in deposition of these elements onto the photocathode surface. Their thicknesses were determined based on XPS analyses (see Supplementary Material). The average Cs(Te) thickness for photocathode (A) was 0.8(8.3) Å and for photocathode (B) 2.9(11) Å. Furthermore, a slight oxygen content equivalent to a layer of 0.5–1 Å was detected. The Cs surface content is comparable for the treated and untreated regions of the individual photocathodes and is expected to result in a reduction of the surface work function, while the presence of Te and O on the cathode surface is only expected to result in a reduction of the QE as they slightly increase the effective work function *W* [59]. We also assume that the small magnitude of these parasitic layers in the sub-nm scale should not alter the plasmonic response of the nanostructures significantly, but we note that in general the photoemission rate and transition absorption for nanoparticles surrounded by various media benefits from surrounding them with materials with lower permittivity [60].

The photocathodes were transferred to the photocathode characterization beamline using a load-lock system. The gun was maintained at a constant base pressure of less than 10^−10^ mbar and a voltage of 65 kV produced a field of 6.5 MV/m at the cathode surface. The electron beam was produced employing a linearly polarized DUV laser beam at *λ* = 266 nm, 5 ns pulse duration, and 10 Hz repetition rate and directed to the photocathodes via in-vacuum mirror with an incidence angle of 5° with respect to the surface normal. The focused spot size was of approximately 1.5–2 mm FWHM at the photocathode surface. The DUV laser beam was then rastered over the photocathode surface using a motorized mirror while simultaneously measuring the generated charge for different values of the pulse energy. Finally, four solenoids (S_1_ – S_4_) provided weak focusing along the electron beamline and the electron bunch charge was monitored simultaneously by a wall-current monitor (WCM) and a Faraday cup.

**Figure 4:**
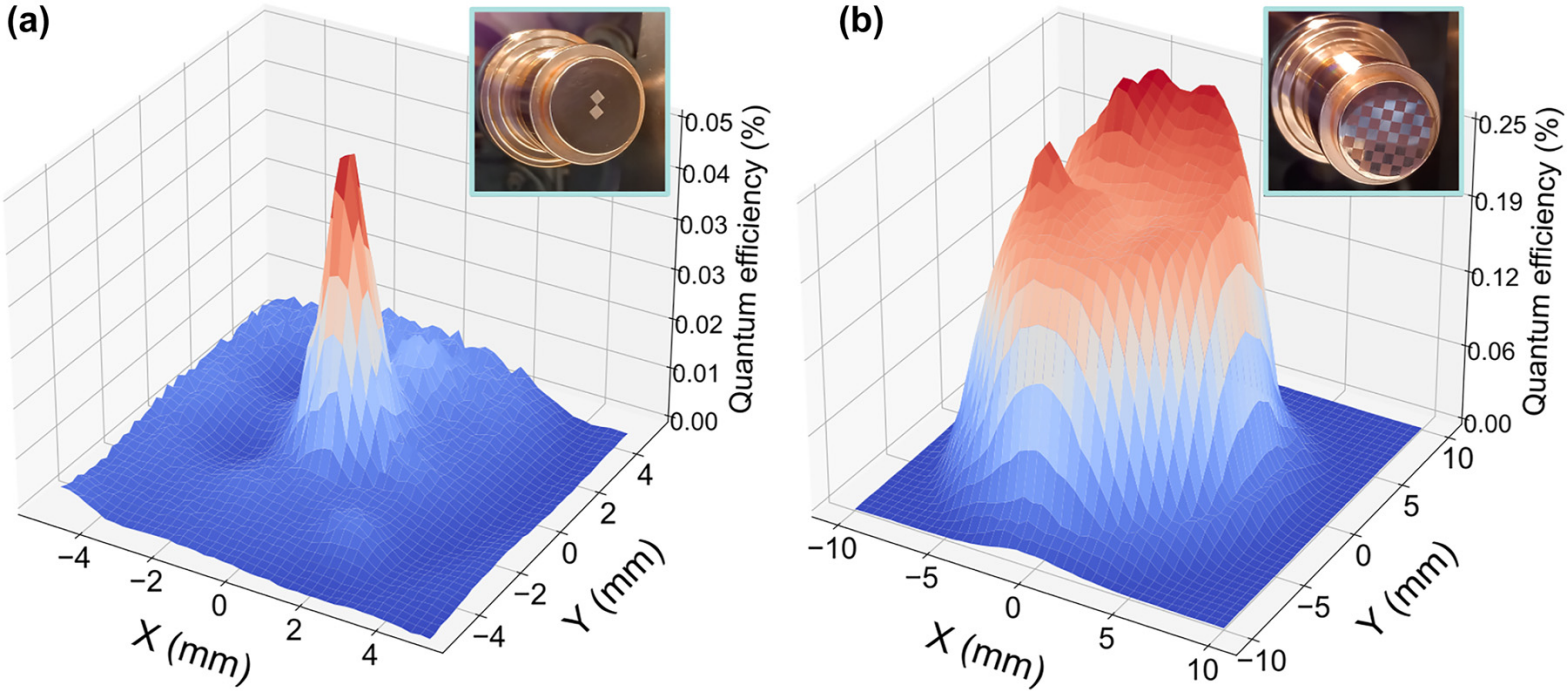
Spatially resolved quantum yield of nanostructured photocathodes. (a) QE map obtained for photocathode (A) using a pulse energy of 5.71 µJ. (b) QE map obtained for photocathode (B) using a pulse energy of 13.34 µJ. The quantum efficiency was calculated by scanning the surface of each of the photocathodes with a step size of 0.25 mm and 0.5 mm, respectively. Each step consisted in an average of 100 consecutive readings of the QE under a repetition rate of 10 Hz.

The experimental results are presented in Figure 4. The charge produced from the untreated flat copper surface of photocathode (A) resulted in a QE of approximately 9.95 × 10^−5^. In contrast, the QE in the nanostructured area was measured to be approximately 4.45 × 10^−4^, showing an enhancement factor 4.5, as shown in Figure 4(a). The photocathode (B) QE analysis retrieved the results shown in Figure 4(b). In this case, the maximum measured QE was 2.58 × 10^−3^, which is approximately an enhancement factor of 26 when compared to the untreated area. As stated before, the laser spot size did not allow to spatially resolve the squared nanopatterned areas, and so our QE results were an average over a surface of around 3–4 mm^2^, therefore the maximum QE enhancement in the case of photocathode (A) is likely to be higher than the reported here. During the fs-laser nanopatterning process, additional debris particles [61] were re-deposited in the vicinity of the treated areas, smearing further the spatial resolution of the QE measurement. In all cases, the measured QE was insensitive to the polarization state of the impinging DUV laser indicating a rotational symmetry of the nanoparticle morphology and spatial distribution, thus ruling out any contribution from the ripple-like patterns. The summary of the main results is presented in Table 1.

**Table 1:** Summary of the results obtained with each photocathode. The QE enhancement factors are calculated by comparing with the flat areas of photocathode (A).

Photocathode	Nanoparticle *r*	Cs thickness	max{QE} (enhancement)
(A) Untreated	–	0.8 Å	9.95 × 10^−5^ (−)
(A) Treated	49 ± 19 nm	0.8 Å	4.45 × 10^−4^ (4.5 × )
(B) Treated	32 ± 8 nm	2.9 Å	2.58 × 10^−3^ (25.9 × )

**Figure 5:**
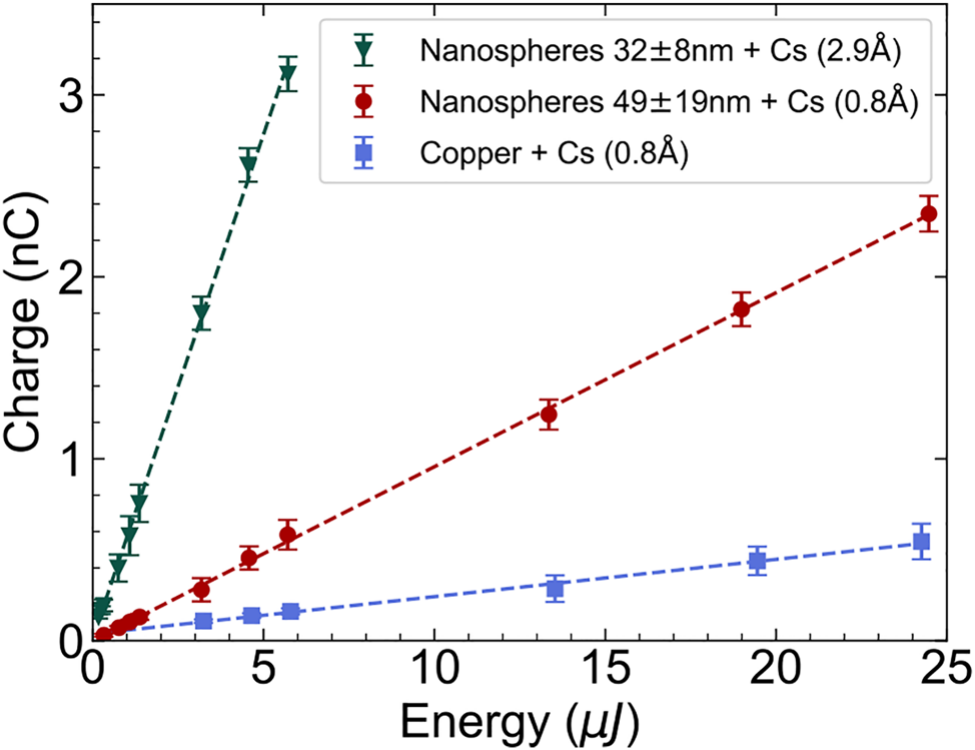
Measurements of the charge generated as a function of the delivered UV pulse energy at the center of the photocathode (B) surface, the nanostructured area of photocathode (A) and the flat area of photocathode (A). The QE is calculated with the slope of the fitted linear trends of photoemission.

Figure 5 shows the produced bunch charge as a function of DUV pulse energy in each area of interest for both photocathodes. Here, the produced charge was always kept below 3.5 nC to avoid saturation effects in the different charge monitoring devices used. The bunch charge shows a linear trend with DUV pulse energy, suggesting that the photoemission process was linear and therefore space charge and other non-linear photoemission effects could be neglected. The plasmonic effect was expected to scale linearly with the applied laser field, which is consistent with the experimental results. The measured QE value for the untreated copper in photocathode (A) is close to that reported in the literature at this wavelength and under similar electrostatic extraction potential (3 × 10^−5^ – 2 × 10^−4^) [62], suggesting that a Cs thin-film of 0.8 Å in thickness did not contribute significantly to the QE. Given the similarity in the chemical content and composition between treated and untreated areas as measured by XPS, we suggest that the enhancement of the QE in photocathode (A) could be originated exclusively from the interaction between the DUV light and the morphological features present in the nanostructured areas.

Photocathode (B) presents a Cs content significantly higher (a factor of 3.5 times) than that measured for photocathode (A) and equivalent to a layer thickness of 2.9 Å. It is well known that cesiated copper surfaces can have a reduced work function down to 1.8 eV [63]. Following Fowler-Dubridge theory, such a decrease in work function translates into a direct increase of QE, given that *QE* ∝ (*ℏω* − *ϕ*)^2^ when the temperature dependence of the photoemission process is small. Here *ϕ* is the height of the emission barrier above the Fermi level (work function *W* minus Schottky barrier lowering factor) [64, 65]. The reduction in work function can be accurately calculated using the Gyftopoulous-Levine formalism as a function of caesium monolayer coverage. Following this theory, the optimal Cs film thickness over a copper substrate is around 6–8 Å [66], which is significantly larger than the estimated here via XPS measurements. Moreover, the photocathode (B) thin-film coverage is less than 20 % mono-layer, and negligible for the case of photocathode (A). Nevertheless, unlike in photocathode (A), the increase in QE of photocathode (B) can be associated with the composite contributions of the Cs_*x*_Te_*y*_ layer and the plasmonic effect in the nanoparticles.

## 5 Conclusions

To conclude, we show that disordered nanostructures and nanospheres featuring plasmonic enhancement can effectively aid in the production of high charge electron beams from metallic photocathodes, specifically on copper, which is common in photoinjectors worldwide. The measurement of the charge generated as a function of laser pulse energy over the produced photocathodes suggests that hot electrons may play an important role in the resulting QE when directly comparing the emission from plasmonic nanostructures and flat surfaces with nearly identical chemical composition. The experimental results showed significant QE enhancement factors between 5 and up to 25. We believe that this approach could be very promising for improving current photocathode technology, specially considering that further optimization of the nanostructures can be easily achieved with commercial ultrafast laser technology.

Our fabrication method is highly versatile, single step, easy to integrate in most electron guns and can be easily tuned to specific material properties and photoemissive parameters. Compared to other nanofabrication techniques, our approach results in rugged and durable nanostructured surfaces, with the capability of *in-situ* rejuvenation. We also show that the charge may be further increased depositing caesium and tellurium over the copper nanostructures, which could also be implemented at the gun level with dispensers. Further experiments with optimized semiconductor layers should be performed to fully understand the potential of this approach, as well as measurements of the emittance of the produced electron beams. Nevertheless, our results readily open new avenues for metallic photocathode improvement and represent a step further in the understanding of plasmonics effects occurring during photoemission processes, which are highly relevant for advanced electron accelerator development.

## Supplementary Material

Supplementary Material Details
