# Supplementary material for: Hot electron enhanced photoemission from laser fabricated plasmonic photocathodes
Source: Nanophotonics. 2023 Oct 17;13(11):1975–83. doi: 10.1515/nanoph-2023-0552 (PMC11501739; doi:10.1515/nanoph-2023-0552)
Supplement: Supplementary file 1 — Supplementary Material Details [file j_nanoph-2023-0552_suppl_001.pdf]

Supporting information: Hot electron enhanced photoemission from laser fabricated plasmonic photocathodes

M. Martinez-Calderon[1]*, B. Groussin[1], V. Bjelland[1,2],†, E. Chevallay[1], V. N. Fedosseev[1], M. Himmerlich[1], P. Lorenz[3], A. Manjavacas[4], B. Marsh[1], H. Neupert[1], R. E. Rossel[1], W. Wuensch[1], and E. Granados[1]**

[1]CERN, European Organization for Nuclear Research, 1211 Geneva, Switzerland
[2]Department of Physics, NTNU–Norwegian University of Science and Technology, NO-7491 Trondheim, Norway
[3]Leibniz Institute of Surface Engineering (IOM), Department of ultra-precision surfaces, Permoserstr. 15, 04318 Leipzig, Germany
[4]Instituto de Óptica (IO-CSIC), Consejo Superior de Investigaciones Científicas, 28006 Madrid, Spain

email: * miguel.martinez.calderon@cern.ch, **eduardo.granados@cern.ch

In this document, we provide additional information and data related to the fabrication of the nanostructured photocathodes, as well as their surface composition. The results are accompanied by COMSOL simulations of field enhancement achievable with a variety of copper surface nano-morphologies.

Contents

1 Direct-laser copper nano-processing 1

2 Electric field enhancement simulations 3
2.1 Nanospheres partially immersed in Cu surfaces . . . 3
2.2 Periodic nano-grooves . . . 5

3 Surface analysis using X-ray photoelectron spectroscopy 5

4 DC gun setup details 6

1 Direct-laser copper nano-processing

The photocathodes used in this work consist of oxygen-free electronic (OFE) grade copper photocathode plugs (with oxygen content $< 5\times10^{-4}$). The Copper photocathode plugs are turned and polished with diamond powder in order to achieve an Ra < 0.02 µm on the substrate surface. The top surface has 19 mm diameter and further details about the plugs design and dimensions can be found in [1]. Before and after laser processing, the CERN standard procedure for UHV cleaning which consists in wet-chemically degreasing with a commercial detergent and subsequent rinsing in deionized water was applied to both photocathodes [2].

Two different ultrafast laser systems were used for photocathode (A) and (B) surface nanopatterning. Laser parameters sweeps (fluence, scanning speed, spot size) were carried out with both systems to find optimum nanostructuring on test samples made from the same type of copper and using the same polishing and cleaning procedures as for the copper photocathode plugs. After SEM analysis of the different structures generated by each set of laser parameters, optimal conditions were replicated on the final copper photocathode (A) and (B) plugs. In the next paragraphs, the final fabrication parameters for each photocathode together with a detailed description of each ultrafast laser system are described.

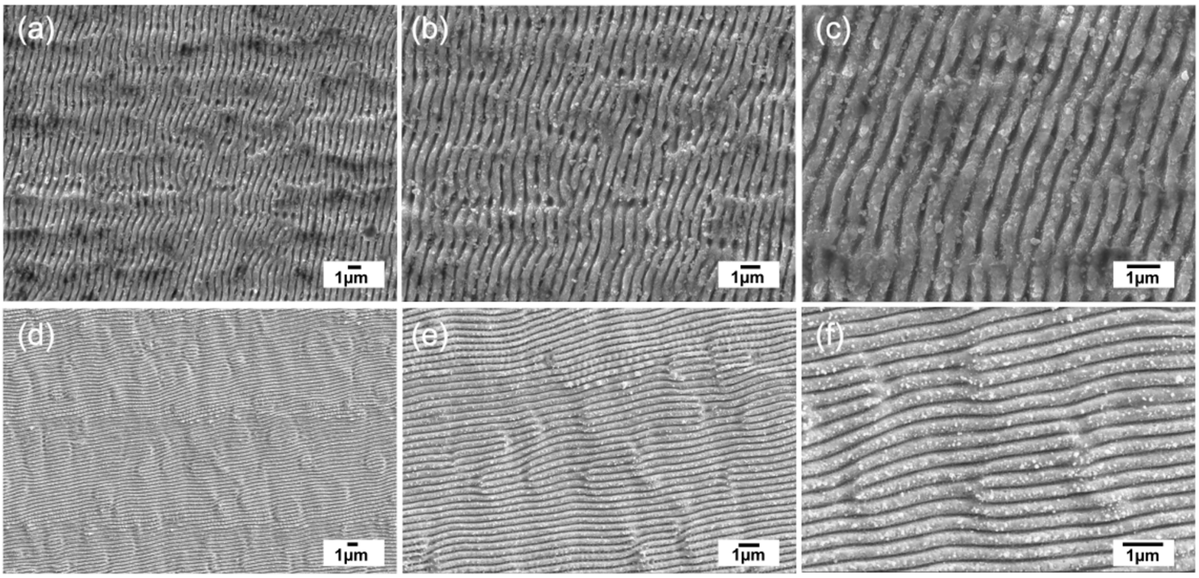

Figure S1: SEM images at different magnifications of the nanostructures fabricated at the surface of: (a, b, c) photocathode (A) and (d, e, f) photocathode (B).

The nanopatterning of photocathode (A) was carried out with a Ti:sapphire laser system consisting of a mode-locked oscillator and a regenerative amplifier (Coherent Libra™). The system delivered up to 2 mJ, 130 fs pulses at a central wavelength $\lambda = 800$ nm, with a 1 kHz repetition rate. The laser power was adjusted to 0.2 mW with a two-step setup: a variable attenuator formed by a half-wave plate and a low dispersion polarizer and neutral density filters. The initial 5 mm laser beam diameter was focused onto the copper samples by means of a broadband 10× microscope objective with a NA of 0.16. The focused beam spot at the samples surface was measured using a 50× microscope objective and a Coherent LaserCam™HR-UV, yielding approximately a beam waist ω_0 of 5.5 µm. The laser polarization was set parallel to the scanning direction and a three-dimensional translational stage was used to move the sample under the laser beam with a velocity of 70 µm/s. Figure S1(a, b, c) shows SEM images of the nanostructures produced with the selected set of laser parameters.

The final 4 mm^2 nanopatterned areas were produced by 1D line-scanning of the laser spot with the described parameters using a separation distance between the lines of 2.5 µm. Two nanopatterned areas of 4 mm^2 were produced at the center of photocathode (A) and the rest of the surface was not irradiated as shown in Fig. S2(a, b).

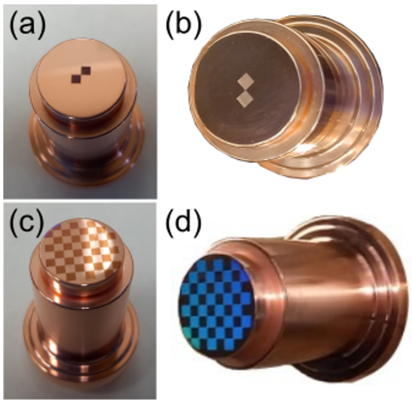

Figure S2: Photographs of the produced nanostructured photocathodes: (a, b) corresponding to photocathode (A). (c, d) corresponding to photocathode (B).

The nanopatterning of photocathode B was performed by a fs-laser system (Light Conversion Pharos™) delivering up to 2 mJ at $\lambda = 1030$ nm. The output beam was frequency-doubled to $\lambda = 515$ nm, the power was adjusted to 90 mW with a pulse duration of 260 fs and a repetition rate set to 100 kHz.

The sample was mounted into a laser material processing system from 3D MicroMac with a computer-controlled x-y stage. The laser beam was focused by a f-theta lens with 165 mm focal length onto the sample surface with a ω_0 of 14 µm determined by Liu Plot [3]. The final 4 mm^2 nanopatterned areas were produced by 1D line-scanning of the laser spot through a galvanometer, setting a scanning velocity of 10 mm/s and a separation distance between the lines of 10 µm. Figure S1(d, e, f) shows SEM images of the nanostructures produced with the selected set of laser parameters.

The controlled movement of the laser beam over the whole photocathode (B) surface allowed the production of irradiated and non-irradiated 4 mm^2 areas with a chessboard-like structure, as shown in Fig. S2(c, d).

2 Electric field enhancement simulations

The objective of the simulations is to examine the response of three-dimensional copper morphologies present in the experimentally tested samples when exposed to a DUV laser beam perpendicular to them. By roughly tailoring the surface structures in size and shape, it is possible to induce the excitation of localized surface plasmons. These excitations produce an enhancement of the amplitude of the laser field E_0 by a factor denoted as $g = |E_s|/|E_0|$, where E_s represents the local electric field computed at each point on the metallic surface.

We perform electromagnetic simulations using COMSOL™ to determine the expected value of g for various geometries and wavelength parameters. We employ the Finite Element Method (FEM) solver to solve Maxwell's equations within a three-dimensional domain. The simulation first focuses on a thick copper (Cu) surface situated in vacuum. The dielectric properties of copper are modeled using $\varepsilon = 1$ (typical for standard metals) and $\sigma = 5.998\times10^7$ S/m. The simulations were also performed with other values for the dielectric loss taken from [4] given that the illumination was at DUV wavelengths, although the results for the field enhancement varied less than 1%. For all simulations, the system is excited with an incident electromagnetic plane wave, linearly polarized, defined as $E = E_0 e^{-ikz}\hat{y}$, with $E_0 = 1$ V/m and $k = 2\pi/\lambda$ (where λ represents the wavelength). The FEM meshing refinement is set to a precision level of $\lambda/10$, and a parametric sweep is employed in the solver to comprehensively examine the behavior of the plasmonic system.

2.1 Nanospheres partially immersed in Cu surfaces

We place a Cu nanosphere of variable radius R on a Cu surface with a penetration depth inside the surface set at $0.2 \times R$ to match roughly the experimental observation using SEM microscope images. The study is conducted looking at variations of the (R, λ) pairs. Due to the rotational symmetry of the simulated arrangement, the polarization orientation does not play any role.

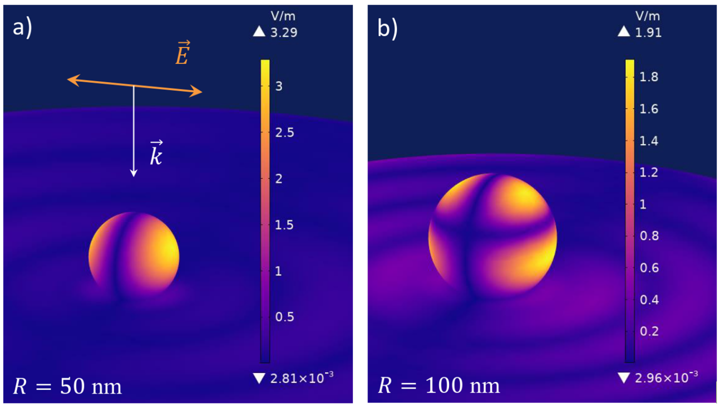

Figure S3: Visualization of field enhancement in partially immersed Cu nanospheres with radii (a) 50 nm and (b) 100 nm when irradiated with 266 nm light.

Figure S3 shows the surface enhancement achievable for two different nanosphere radii. The 50 nm radius nanosphere excited by a 250 nm incident field is expected to display an enhancement with $g > 3.5$, as shown in Fig. S3a. For larger particle sizes, plasmonic behavior is also clearly observed, as shown Fig. S3b. However, it corresponds to higher-order plasmonic resonances, consequently yielding a lower

maximum electric field enhancement factor, just above 1.8. Note that here we study the surface field data only and not the surrounding field.

By varying both the radius and the wavelength we can now compute an overall map displaying the plasmonic resonances of the system. The corresponding results are shown in Fig. S4. They portray the possibility of enhancing the field up to a factor of 3.5 by roughly matching the nanosphere radius to 1/6 of the incident wavelength. The broad resonance of the nanospheres is also an indicator of their low quality factor, although also points at a low requirement for size accuracy. This enables us to use laser techniques for fabricating resonant nanospheres with loosely matched size but still enhancing the field relatively efficiently.

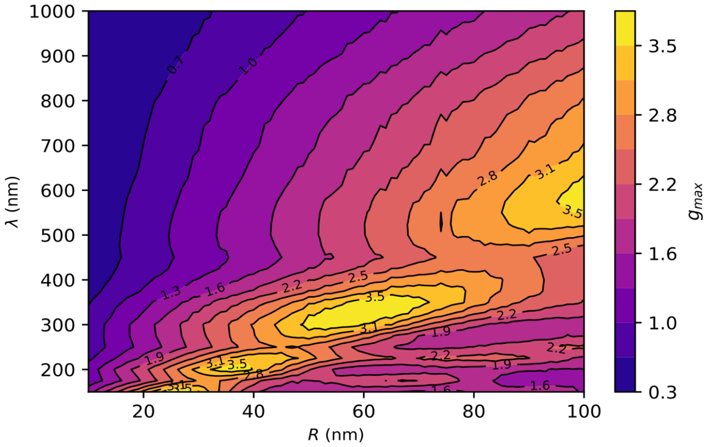

Figure S4: Overall maximum field enhancement factor achievable at the surface of partially immersed Cu nanospheres with various radius R and at a range of excitation wavelengths from 200 to 1000 nm.

Figure S5 depicts the vector components of the amplified field within the polarization plane near the nanosphere. The size and direction of the arrows indicate the intensity and orientation of E at each specific point. Our observations reveal that while the surface field enhancement in the metal reaches a maximum of $g = 3.5$ for a 50 nm sphere, in the immediate vicinity of the surface, it rises to $g > 6$. The orientation of the field varies depending on the plasmonic lobes associated with different plasmonic modes, highlighting the significance of the phase of the wave E. The plasmonic field exhibits alternating positive and negative orientations relative to the surface of the sphere, oscillating over time.

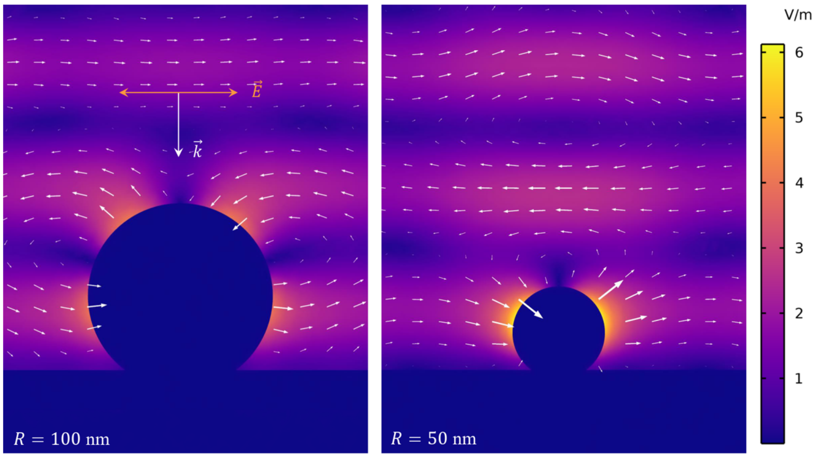

Figure S5: Overall maximum field enhancement factor achievable at the surface of partially immersed Cu nanospheres with various radius R and at an excitation wavelength of 266 nm.

2.2 Periodic nano-grooves

The SEM images clearly revealed the presence of periodic ripples known as laser induced periodical surface structures (LIPSS) [5], with periodicities ranging from approximately 380 nm to 570 nm. In our modelling approach, we construct semi-cylinders that are periodically placed on a surface, with smaller semi-cylindrical grooves carved into the surface to separate them. The aspect ratio between the cylinder and groove was established as 1/5 based on observations from SEM images and profilometric studies as shown in Figure S6a. To maximize the field enhancement factor, the structures were oriented perpendicular to the polarization of the incident wave. Figure S6a shows the results of field enhancements for a periodicity of 170 nm, which was best matched to DUV illumination wavelengths to gain insight in the enhanced plasmonic properties when illuminated in DUV laser driven photoinjectors. For larger periodicities the enhancement was rather negligible at this wavelength.

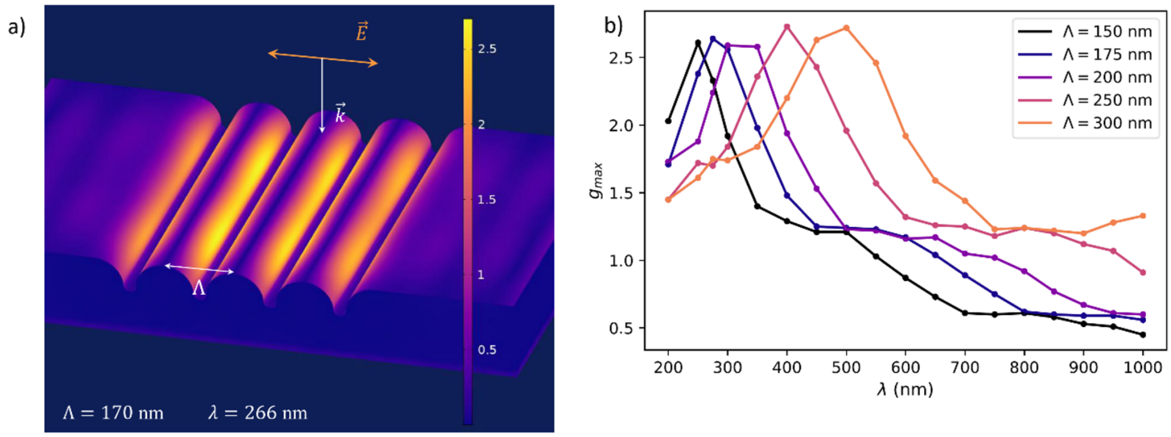

Figure S6: (a) Field enhancement in the vicinity of the nanogrooves with spatial periodicity of 170 nm when illuminated with 266 nm pulses. (b) Calculated maximum field enhancement as a function of excitation wavelength for various ripple periodicities.

The arrays of cylindrical structures exhibit evident plasmonic behavior, with both the position and intensity of the field enhancement varying with the excitation wavelength. In order to maximize the plasmonic resonance, the orientation of the array was set perpendicular to the polarization of the incident field. This configuration was found to result in a more intense plasmonic response compared to when the cylinders were aligned parallel to the incident field. When parallel, only the tips of the cylinders are influenced, resulting in less overall surface area interacting with the plasmon.

In order to understand better the periodicity requirements as a function of excitation wavelength, we explored spatial periodicities Λ going from 150 to 300 when illuminated between 200 and 1000 nm. Figure S6b illustrates the resonance peaks of the structure within the maximum enhancement factor dataset, exhibiting a shift towards longer resonant wavelengths as the spatial periodicity (Λ) increases. This observation is logical as a larger spatial periodicity scales up the characteristic size of the ripples, resulting in larger resonant cavities that require longer wavelengths to resonate. Between 170 nm to 200 nm, the resonant wavelengths are shown to fall between 275 nm and 320 nm, with an enhancement factor $g_{\mathrm{max}} = 2.58$. Although this value is promising, it is important to consider the spatial distribution of the field, which will eventually decrease the effective experimental enhancement. It should be noted that the model used may not be geometrically accurate, and more precise modelling may result in varying and potentially decreased field enhancement values.

3 Surface analysis using X-ray photoelectron spectroscopy

The samples were characterized by X-ray photoelectron spectroscopy following the transfer and using the experimental setup described in [6]. Figure S7 includes the spectra that were acquired on laser-treated and untreated regions of the photocathodes A and B. Since mainly Cs, Te, O and C were detected on the Cu surface, we have modelled the spectra using the NIST Database for the Simulation of Electron Spectra for Surface Analysis (SESSA): Version 2.0 using a Cs_xTe_y layer on Cu with C and O surface

adsorbate layers. The resulting layer compositions and thicknesses (equivalent Cs and Te single layer thicknesses) are:

- Photocathode A:
  – laser-treated: 11.0 Å Cs_1Te_{30} (0.8 Å Cs & 8.3 Å Te) with 1.0 Å C and 1 Å O
  – untreated: 11.0 Å Cs_1Te_{30} (0.8 Å Cs & 8.3 Å Te) with 1.0 Å C and 0.5 Å O

- Photocathode B:
  – laser-treated: 15.0 Å Cs_1Te_6 (2.5 Å Cs & 11.0 Å Te) with 1.0 Å C and 0.5 Å O
  – untreated: 14.5 Å Cs_1Te_7 (3.3 Å Cs & 11.1 Å Te) with 1.0 Å C and 0.5 Å O

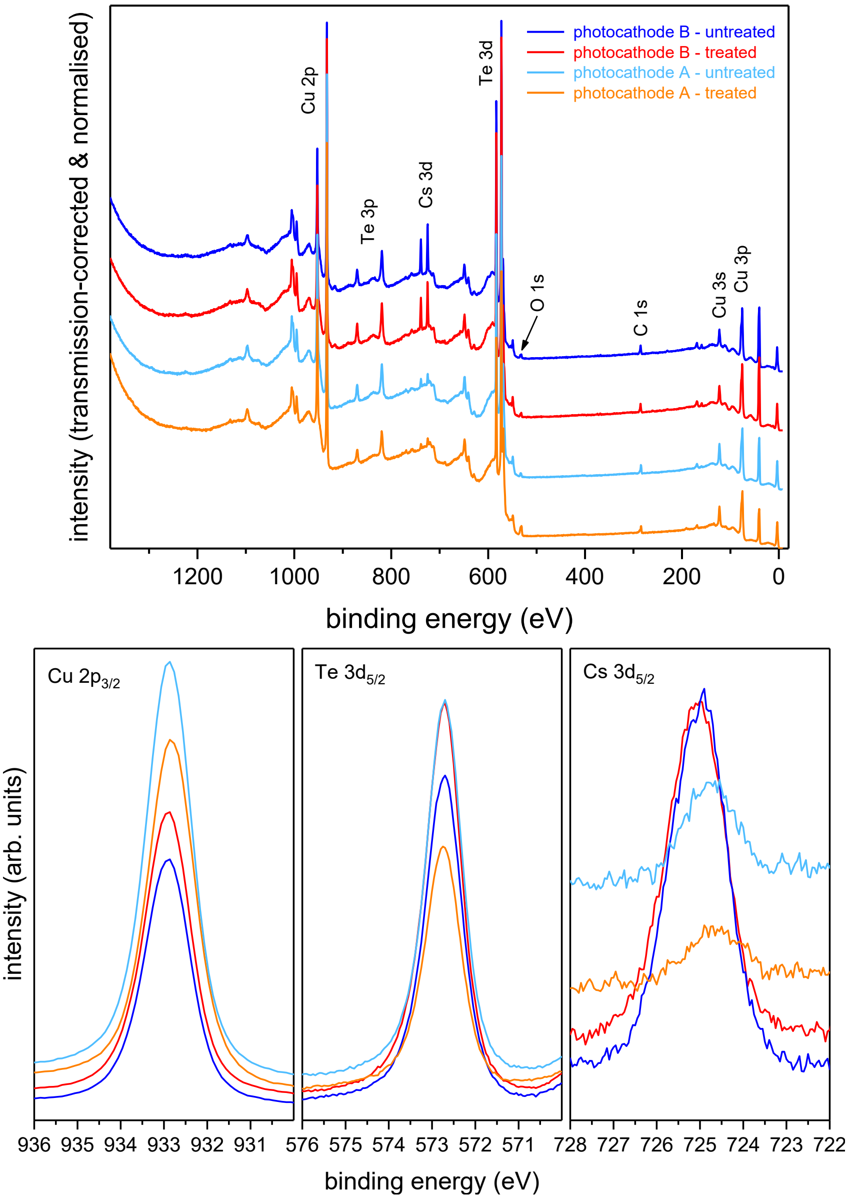

Figure S7: X-ray photoelectron spectra of the laser-treated and untreated regions of photocathodes A and B (top: survey spectra, bottom: detail spectra of the Cu $2p_{3/2}$, Te $3d_{5/2}$ and Cs $3d_{5/2}$ states).

4 DC gun setup details

After the nanopatterning and the cleaning processes, the photocathodes were initially introduced into the DC-GUN preparation chamber fully opened and exposed to air atmosphere. Then, Ultra High Vacuum

(UHV) conditions were reached in the preparation chamber following three main steps. First, an initial vacuum in the range of 10^{-7} - 10^{-8} mbar was established through primary and turbo pumps. Second, a typical bake-out process ramping up the temperature to 150 - 250 degrees (depending on the heated element) was performed. Third, after ramping down to room temperature, ion and sublimation pumps were activated achieving a constant base pressure in the range of 10^{-11} mbar. Pirani and penning gauges are present in each section of the setup to monitor the corresponding pressures and a residual gas analyzer is used to monitor gaseous contaminants.

Once under UHV, the preparation chamber is used either for storage under UHV or thin-film thermal evaporation of photo-emissive layers such as Cesium, Tellurium or Antimony [7]. Otherwise, the photocathodes are transferred under vacuum to the DC-gun testing position using a photocathode manipulator (both setup sections are separated by a UHV mechanical stainless-steel valve).

The DC-gun is operated at a nominal voltage of 65kV leading to a 6.5 MV/m electric field at the photocathode surface. For proper transport of the electron beam that exits the gun, four solenoidal magnets S_1–S_4 are employed along the beamline for weak focusing, as shown in the manuscript Fig. 3. A wall-current monitor (WCM) with an acceptance bandwidth from 1 MHz to 10 GHz [8] located between S_1 and S_2 (see Fig. 3 in the manuscript) is used to measure the electron beam current as well as a Faraday cup (FC) which is located at the end of the beamline.

For electron beam production, the light source consisted in a laser system delivering 5 ns pulses centered at $\lambda = 1064$ nm with 10 Hz repetition rate. The output was frequency-doubled to 532 nm and then frequency-quadrupled for a final wavelength $\lambda = 266$ nm (4.66 eV). The polarization was controlled through a half-wave plate. The beam was focused onto the photocathodes surface by means of a keplerian telescope (with two lenses of focal length 100 and 150 mm) and a focusing lens with 3 m focal length, leading to a focused beam diameter at the photocathode surface of approximately 2.5 mm FWHM.

The UV beam energy delivered to the photocathode was online monitored during all the tests by sampling a portion of the beam before the scanning translation stages and regulated using UV neutral density filters allowing values from 0.1 to 160 µJ at the photocathodes surface. For further monitoring and initial alignment of the UV beam at the center of the photocathode surface, a virtual cathode line was used. The virtual line matches the distance from a beam sampler before the beam enters vacuum, to the photocathode surface and allows to monitor the UV beam position and profile using a scintillating screen imaged by a CCD camera.

The UV beam was introduced into the DC-gun setup through a UV viewport and then deflected to the photocathodes surface with a 5 degrees angle of incidence with an in-vacuum UV mirror. The beam was x-y scanned along the whole photocathodes surface through the movement of one of the mirrors mounted onto two translation stages allowing a spatial resolution of approximately 1 µm.

References

[1] Irene Martini. Characterization of Cs-Sb cathodes for high charge RF photoinjectors, 2016. Presented 19 Feb 2016.

[2] M. Malabaila. Procédure de dégraissage avec la machine aux solvants MEG, 2017. https://edms.cern.ch/document/1810387/1.

[3] J. M. Liu. Simple technique for measurements of pulsed gaussian-beam spot sizes. *Opt. Lett.*, 7(5):196–198, May 1982.

[4] P. B. Johnson and R. W. Christy. Optical constants of the noble metals. *Phys. Rev. B*, 6:4370–4379, Dec 1972.

[5] Jörn Bonse and Stephan Gräf. Maxwell meets marangoni—a review of theories on laser-induced periodic surface structures. *Laser & Photonics Reviews*, 14(10):2000215, 2020.

[6] H. Panuganti, E. Chevallay, V. Fedosseev, and M. Himmerlich. Synthesis, surface chemical analysis, lifetime studies and degradation mechanisms of cs-k-sb photocathodes. *Nuclear Instruments and Methods in Physics Research Section A: Accelerators, Spectrometers, Detectors and Associated Equipment*, 986:164724, 2021.

[7] E. Chevallay. Experimental results at the CERN photoemission laboratory with co-deposition photocathodes in the frame of the CLIC studies. Technical report, CERN, Geneva, 2012. https://clic-study.web.cern.ch/sites/default/files/pdfs/notes/CTF3Note104.pdf.

[8] J Durand, T Tardy, and M Wurgel. A 10 GHz wall current monitor. Technical report, CERN, Geneva, 1995.

Supporting information
